# Development and Characterization of Transcription Factor Gene-Derived Microsatellite (TFGM) Markers in *Medicago truncatula* and Their Transferability in Leguminous and Non-Leguminous Species

**DOI:** 10.3390/molecules20058759

**Published:** 2015-05-15

**Authors:** Wenxian Liu, Xitao Jia, Zhimin Liu, Zhengshe Zhang, Yanrong Wang, Zhipeng Liu, Wengang Xie

**Affiliations:** State Key Laboratory of Grassland Agro-ecosystems, College of Pastoral Agricultural Science and Technology, Lanzhou University, Lanzhou 730020, China

**Keywords:** *Medicago truncatula*, transcription factor, microsatellite, transferability

## Abstract

Transcription factors (TFs) are critical adaptor molecules that regulate many plant processes by controlling gene expression. The recent increase in the availability of TF data has made TFs a valuable resource for genic functional microsatellite marker development. In the present study, we developed TF gene-derived microsatellite (TFGM) markers for *Medicago truncatula* and assessed their cross-species transferability. A total of 203 SSRs were identified from 1467 *M. truncatula* TF coding sequences, 87.68% of which were trinucleotide repeats, followed by mono- (4.93%) and hexanucleotide repeats (1.48%). Further, 142 TFGM markers showed a high level of transferability to the leguminous (55.63%–85.21%) and non-leguminous (28.17%–50.00%) species. Polymorphisms of 27 TFGM markers were evaluated in 44 alfalfa accessions. The allele number per marker ranged from two to eight with an average of 4.41, and the PIC values ranged from 0.08 to 0.84 with an average of 0.60. Considering the high polymorphism, these TFGM markers developed in our study will be valuable for genetic relationship assessments, marker-assisted selection and comparative genomic studies in leguminous and non-leguminous species.

## 1. Introduction

Evaluation and understanding of the genetic variation within the germplasm collection by using molecular markers is crucial for the effective conservation and use of genetic resources [1]. Microsatellites or simple sequence repeats (SSRs) are PCR-based, multi-allelic, co-dominant genetic markers consisting of 1‒5 nucleotide core units that are tandemly repeated. Because of their desirable genetic attributes, including hypervariability, co-dominant heritability, reliability, wide genomic distribution, chromosome-specific location and being multi-allelic, SSR markers have become the marker class of choice for population diversity studies, genetic map construction, marker-assisted selection and gene mapping [2]. SSR markers can be developed from either genomic libraries or public databases, such as expressed sequence tags (ESTs) and transcriptome sequences [3]. The development of SSR markers from genomic libraries is expensive and inefficient. Compared to genomic SSRs, SSRs markers derived from ESTs, particularly with well-characterized function genes, are expected to contribute to metabolism and gene evolution, which makes them act as “functional genetic markers” for rapidly establishing marker-trait linkages and to identify genes/quantitative trait loci (QTLs) for traits of agricultural importance in crop plants [1,4]. In addition, the recent increase in the availability of genome sequences has facilitated the development of SSR markers in ESTs and protein-coding function genes with the help of bioinformatics tools [4].

Transcription factors (TFs) are proteins that play key roles as *trans*-acting factors in the stress response and in plant development by binding to the *cis*-acting elements in the promoters of target genes, and they are assumed to have fundamental roles in the evolution of species [5]. A deep understanding of transcription factors and their regulatory networks would improve the understanding of organism diversity [6]. With the recent advancement of novel array-based sequencing technologies and modern genomic tools, many plant TFs have been identified and annotated [7]. The availability and continuous enrichment of the TFs with well-characterized functional domains could provide excellent candidates and serve as a valuable transcriptomic resource for novel sequence-based genic functional microsatellite marker development [4]. In *Saccharomyces cerevisiae*, SSR markers are overrepresented among open reading fragments (ORFs) encoding for TFs and protein kinases rather than for structural genes, indicating the role of these markers as a factor contributing to the rapid evolution of adaptive phenotypes [8]. To our knowledge, the development of the transcription factor gene-derived microsatellite (TFGM) markers in plants has only been reported in chickpea to date, and those markers have been proved to have great potential in marker-assisted genetic improvement and genotyping applications [4,9].

Based on area harvested and total production, the legume family is the second most important food and forage source after the grass family [10]. The conservation of genome structure among legumes ensures the transfer of technology from more studied legume species such as *Medicago truncatula*, to others [11]. By now, a large set of SSR markers derived from the *M. truncatula* genomic or EST sequences has been developed and used in legume crops both within and outside the *Medicago* genus [12,13,14]. However, no studies have yet specifically addressed the development and usage of TFGM markers of *M. truncatula*. Previous studies have shown that the results of SSR identification and primer design were related to the search criteria and sequence type [9,12], and the development of new functional microsatellite markers with relatively high polymorphic potential based on *M. truncatula* full-length TF coding sequences will be essential and useful in various applications of genetics, genomics and breeding programs. Therefore, this study was undertaken with following objectives: (1) to analyze the frequency and distribution of SSRs in the *M. truncatula* TFs; (2) to develop and characterize *M. truncatula* TFGM markers, and (3) to assess their cross-species transferability. These TFGM markers developed in our study will be valuable for genetic relationship assessments, marker-assisted selection and comparative genomic studies in leguminous and non-leguminous species.

## 2. Results and Discussion

### 2.1. Frequency and Distribution of SSRs in the M. truncatula TF Genes

In the present study, a total of 1467 TF coding sequences of *M. truncatula* with an average length of 1078 bp were mined for SSRs and used to design the TFGM markers (Table 1). These sequences represent approximately 1582.5 kilobases (kb) of 59 *M. truncatula* transcription factor families, with the number of genes per family ranging from 1 (VOZ, Whirly, LFY, and BES1) to 117 (ABI3VP1) (Figure S1). The *MISA*-based microsatellite search of these TF genes detected a total of 203 SSRs in 176 (12.0%) TF genes, with a distribution frequency of one SSR locus per 7.8 kb, which was similar to the early reports on TF-derived SSRs in chickpea (7.1 kb) [4] and EST-derived SSRs in alfalfa (7.7 kb) [15], peanut (7.3 kb) [16], and sweet potato (7.1 kb) [17] but lower than EST-derived SSRs in *M. truncatula* (1.8 kb) [12], coffee (2.16 kb) [18], and tea (3.5 kb) [19].

**Table 1:** Summary of SSR search results.

Search Items	Numbers
Total number of TFs examined	1467
Total number of identified SSRs	203
Number of SSR containing TFs	176
Number of TFs containing more than 1 SSR	21
Number of SSRs present in compound formation	10
Repeat type	
Mononucleotide	10
Dinucleotide	1
Trinucleotide	178
Tetranucleotide	1
Pentanucleotide	0
Hexanucleotide	3
Total length of sequences searched (kb)	1582.5
Frequency of SSRs	One per 7.8 kb

The *M. truncatula* TF-derived SSRs contained diverse types of repeat motifs, and there was an uneven distribution of SSRs among motif type and location (Table 1 and Table S1). Analysis of SSR motifs in the SSR-containing TF genes revealed that 21 (11.93%) TF genes contained more than one SSR. Of the 203 total SSRs, 193 (95.07%) contained simple repeat motifs, while 10 (4.93%) were compound motifs. Among the different types of simple repeat motifs, trinucleotide motifs were the most abundant (87.68%), followed by mono- (4.93%) and hexanucleotide motifs (1.48%). Only one dinucleotide motif (GA/TC) and one tetranucleotide (GAAA/TTTC) motif were detected, and no pentanucleotide motifs were found in any of the *M. truncatula* TF sequences. Previous studies have shown that trinucleotide repeats were the most common motif for SSR markers developed in many species, followed by either dinucleotide repeats or tetranucleotide repeats [1]. Among cereal species, trinucleotide repeats were the most frequent motif present in the ESTs (54%–78%), followed by dinucleotides (17.1%–40.4%) and tetranucleotides (3%–6%) [20]. Yu *et al.* [21] reported that in wheat, 74% of the trinucleotide repeats were found in coding regions, whereas most of the dinucleotide repeats (81%) were in noncoding regions. However, the most abundant repeat type in *M. truncatula* ESTs was mononucleotide (82.6%), followed by trinucleotide (11.4%), and dinucleotide (4.4%) [12]. In this study, the abundance of trinucleotide repeats in the ORF of *M. truncatula* TF genes could be attributed to the absence of frameshift mutations in coding regions when there is length variation in these SSRs [1].

### 2.2. Functional Classification of SSR Containing TF Genes

**Figure 1:**
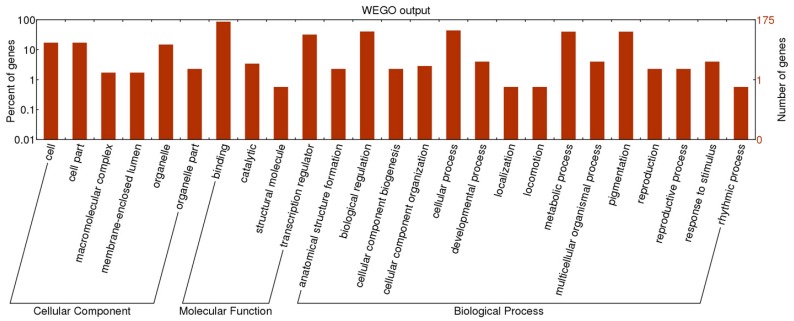
GO classifications of SSR containing transcription factor genes.

To evaluate the potential functions of the SSR containing TF genes, Blast2GO and WEGO software were used to annotate the 176 SSR containing TF genes by searching against GO database. Figure 1 wholly summarizes the categorization of these TF genes according to biological process, cellular component and molecular function. A total of 175 TF genes were finally divided into 25 GO categories. In the biological process category, the two most over-represented GO terms were cellular process (77 genes, 44.0%) and biological regulation (71 genes, 40.6%), followed by metabolic process and pigmentation (both 70 genes, 40.0%). Categories based on molecular function classified the TF genes into 4 groups: 151 TF genes (86.3%) were assigned to binding, followed by transcription regulation (56 genes, 32.0%), catalytic (6 genes, 3.4%) and structural molecule (1 gene, 0.6%). Based on cellular component categorization, cell and cell part genes (30 genes, 17.1% for both) dominated, followed by organelle (26 genes, 14.9%).

### 2.3. Development of M. truncatula TFGM Markers

Of the 176 SSR-containing TF genes, a total of 184 primer pairs could be successfully designed from 160 (90.91%) *M. truncatula* TF genes; the remaining genes either had too-short sequences flanking the SSR loci or did not match the criteria for primer design. Details of the successfully designed primer pairs are provided as supplementary data (Table S1). Of the 184 primer pairs, 167 (90.76%) belong to trinucleotide repeats, and 8 (4.35%), 6 (3.26%), and 3 (1.63%) belong to compound, mononucleotide, and hexanucleotide repeats, respectively.

Based on 3828 EST sequences from *M. truncatula*, 4636 EST-SSR markers have been previously developed [12]. In order to determine whether the 184 TFGM markers developed in this study were novel, the TF sequences used to develop them were cross-referenced with the 3828 ESTs previously reported [12]. The BLASTN results showed that 71 out of 160 TF sequences had significant similarity with 73 EST sequences reported by Gupta *et al*. [12] (Table S2). However, at the SSR loci level, only 54 SSR loci were found to be common (Table S1), meaning that 130 of the 184 (70.65%) TFGM markers developed in our study are novel and may be highly relevant for genetic relationship assessments, marker-assisted selection and comparative genomic studies.

### 2.4. Transferability of M. truncatula TFGM Markers

To assess the cross-species transferability of TFGM markers, 142 *M. truncatula* TFGM markers were tested in two leguminous (alfalfa and chickpea) and three non-leguminous (tobacco, rice, and *Arabidopsis*) species, using *M. truncatula* as a positive control. As shown in Table 2, 123 (86.62%) of the 142 assayed barrel medic TFGM markers provided consistent amplification in barrel medic, 121 (85.21%) in alfalfa, 79 (55.63%) in chickpea, 40 (28.17%) in tobacco, 56 (39.44%) in rice, and 71 (50.00%) in *Arabidopsis*. This result is consistent with a previous study that showed a high cross-species transferability of *M. truncatula* EST-SSR markers across three leguminous species (ranging from 53% to 71%) and three non-leguminous species (ranging from 36% to 44.4%) [12]. The high transferability of *M. truncatula* TFGM markers in leguminous and non-leguminous species indicates that the regions in the TF genes flanking the microsatellites are highly conserved across species [22], which will render these markers useful in the construction of linkage maps and for comparative genomic study and QTL discovery in the future. Furthermore, the transferability of *M. truncatula* TFGM markers in leguminous species is higher than that in non-leguminous species, which is consistent with the general trend of decreasing amplification with increasing evolutionary distances between the species [23].

**Table 2:** Transferability of *M. truncatula* TFGM markers in leguminous and non-leguminous species.

Species	Transferability
Barrel medic	123 (86.62%)
Alfalfa	121 (85.21%)
Chickpea	79 (55.63%)
Tobacco	40 (28.17%)
Rice	56 (39.44%)
*Arabidopsis*	71 (50.00%)

### 2.5. Genetic Diversity Analysis of 44 Alfalfa Accessions

Thirty-five TFGM primer pairs randomly selected from the 121 transferable markers in alfalfa were tested for their potential in genetic studies by ascertaining the genetic diversity in 44 alfalfa accessions (Table 3). The screening results revealed that all primer pairs had reproducible amplifications across the 44 alfalfa accessions and that 27 (77.14%) were polymorphic (Table 4). A total of 119 alleles were detected from the 27 polymorphic TFGM markers, and 78 of these alleles were polymorphic. The number of alleles produced per primer pair ranged from two (MtTF14, MtTF51, and MtTF65) to eight (MtTF19) with an average of 4.41. The highest polymorphism information content (PIC) value was observed with primer MtTF70 (0.84) and the lowest was observed for MtTF64 (0.08), and the average PIC value was 0.60 (Table 4). It has been suggested that PIC values greater than 0.5 indicate informative markers, whereas loci with PIC values greater than 0.7 are suitable for genetic mapping [24]. In the present study, 19 and 10 TFGM markers have PIC values greater than 0.5 and 0.7, respectively, which indicates the high level of polymorphism of these markers and their potential in genetic diversity and genetic mapping analyses.

**Table 3:** List of 44 alfalfa accessions used for genetic diversity analysis in this study.

No.	Name	Species	Type	Country of origin
1	WL363HQ	*M. sativa ssp. sativa*	Cultivar	United States
2	Saranac AR	*M. sativa ssp. sativa*	Cultivar	United States
3	WL343HQ	*M. sativa ssp. sativa*	Cultivar	United States
4	Arc	*M. sativa ssp. sativa*	Cultivar	United States
5	UC-1465	*M. sativa ssp. sativa*	Cultivar	United States
6	Aurora	*M. sativa ssp. sativa*	Cultivar	United States
7	WL168HQ	*M. sativa ssp. sativa*	Cultivar	United States
8	UC-1887	*M. sativa ssp. sativa*	Cultivar	United States
9	Maverick	*M. sativa ssp. sativa*	Cultivar	United States
10	W1319HQ	*M. sativa ssp. sativa*	Cultivar	United States
11	Jindera	*M. sativa ssp. sativa*	Cultivar	United States
12	Vertus	*M. sativa ssp. sativa*	Cultivar	United States
13	Saranac	*M. sativa ssp. sativa*	Cultivar	United States
14	Trifecta	*M. sativa ssp. sativa*	Cultivar	Australia
15	Siriver	*M. sativa ssp. sativa*	Cultivar	Australia
16	Hunterfield	*M. sativa ssp. sativa*	Cultivar	Australia
17	Vernal	*M. sativa ssp. sativa*	Cultivar	Australia
18	Sanditi	*M. sativa ssp. sativa*	Cultivar	France
19	Orca	*M. sativa ssp. sativa*	Cultivar	France
20	WL354HQ	*M. sativa ssp. sativa*	Cultivar	France
21	HunterRiver	*M. sativa ssp. sativa*	Cultivar	Mexico
22	Derby	*M. sativa ssp. sativa*	Cultivar	Netherlands
23	Zhongmu1	*M. sativa ssp. sativa*	Cultivar	China
24	Gongnong2	*M. sativa ssp. sativa*	Cultivar	China
25	Gongnong1	*M. sativa ssp. sativa*	Cultivar	China
26	Gannong4	*M. sativa ssp. sativa*	Cultivar	China
27	Zhongmu4	*M. sativa ssp. sativa*	Cultivar	China
28	Zhonglan1	*M. sativa ssp. sativa*	Cultivar	China
29	Zhongmu3	*M. sativa ssp. sativa*	Cultivar	China
30	Gannong5	*M. sativa ssp. sativa*	Cultivar	China
31	Gannong1	*M. sativa ssp. sativa*	Cultivar	China
32	Ningmu1	*M. sativa ssp. sativa*	Cultivar	China
33	Longdong	*M. sativa ssp. sativa*	Cultivar	China
34	Gannong7	*M. sativa ssp. sativa*	Cultivar	China
35	Xinmu1	*M. varia Martyn*	Cultivar	China
36	Gannong2	*M. varia Martyn*	Cultivar	China
37	Tumu1	*M. varia Martyn*	Cultivar	China
38	Tumu2	*M. varia Martyn*	Cultivar	China
39	Caoyuan2	*M. varia Martyn*	Cultivar	China
40	Xinjiangdaye	*M. sativa ssp. sativa*	Land race	China
41	Weixian	*M. sativa ssp. sativa*	Land race	China
42	Wudi	*M. sativa ssp. sativa*	Land race	China
43	Tianshui	*M. sativa ssp. sativa*	Land race	China
44	Longzhong	*M. sativa ssp. sativa*	Land race	China

**Figure 2:**
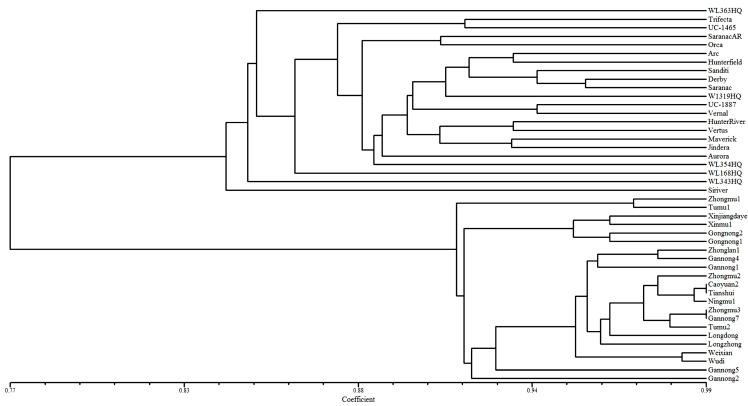
The dendrogram of 44 alfalfa accessions based on UPGMA analysis using 27 polymorphic TFGM markers.

Unweighted pair group method arithmetic mean (UPGMA) cluster analysis was performed to analyze the genetic diversity of 44 alfalfa accessions with the 27 polymorphic TFGM markers. The cluster results showed that the 44 alfalfa accessions could be grouped into two large groups (Figure 2). The first group contained 22 accessions collected from the United States, Australia, France, Mexico, and the Netherlands. The other 22 accessions collected from China, including 17 cultivars and five land races, were clustered into the second group. Although all the indigenous alfalfa accessions could be separated from the exotic accessions and clustered into a single group, the association between the clustering pattern and geographical distribution among the 22 exotic accessions was less significant. Similar results also have been noticed in previous studies [15,25]. The reason for this intermixing of accessions may be due to the small number of the markers or less accessions from each geographical location used in this study. Furthermore, the five *M. varia Martyn* cultivars collected from China were not form separate clusters but scattered among other 17 *M. sativa ssp. sativa* cultivars/landraces, this result might be explained by the recurrent selection methods involving multiple hybridizations and selection activities with available *M. sativa ssp. sativa* and *M. varia Martyn* germplasm in Chinese breeding programs [15]. Nevertheless, the value of the newly developed TFGM markers in our study was emphasized by the results and can be recommended for cultivar identification and assessment of genetic diversity in alfalfa genotypes.

**Table 4:** Details of the 27 polymorphic TFGM markers with their genetic parameter values.

No.	Primer name	Primer sequence (5′-3′)	No. of Alleles	PIC Value	Transcription Factor Family
1	MtTFSSR-1	F: AGCAGCAGGAAACACAGCTT	3	0.59	GRAS
		R: CAATTGGTGAGAGCTGGTGA			
2	MtTFSSR-9	F: TGTTCCATGCAGTAGCTTGC	4	0.67	C2C2_Zn-YABBY
		R: AGGCTGAAATGCTTTGCACT			
3	MtTFSSR-10	F: TAACCCAACTTCCTCAACCG	7	0.72	bHLH
		R: TGCATCAACTCACTTGGCTC			
4	MtTFSSR-14	F: TTTTCGTTGACGACCTCCTT	2	0.34	C2C2_Zn-GATA
		R: GGTCGTTGGTGGGTAGAGAA			
5	MtTFSSR-15	F: ATGCTGCCACCCAAAACTAT	6	0.58	(R1)R2R3_MYB
		R: GAAGCAGAAGAAGAAAATGGGA			
6	MtTFSSR-18	F: GGAAGATCAATGTTGCTGTCAA	6	0.77	C2H2_Zn
		R: AAGGTGGCAAGTTGAGATCG			
7	MtTFSSR-19	F: TTGAGGGTTCAACGTTTGGT	8	0.83	(TAC)5
		R: CTCGAAGCGCGTTAAGAAAC			
8	MtTFSSR-23	F: TCCTTCGCTCTTCGTTGTTT	6	0.78	AP2_EREBP
		R: TCTATGTTGCAGCTGTTGGG			
9	MtTFSSR-24	F: ATCAGCCATGGCATACACAA	5	0.70	WRKY_Zn
		R: TGGTTTGGTGGAATGAAGAA			
10	MtTFSSR-27	F: AATCCACCACCAACAACCAT	4	0.70	C2H2_Zn
		R: GTCCTGTCGGAAACGACCTA			
11	MtTFSSR-28	F: CGGAGAGAATCGAAAGGGAT	5	0.74	C3H-TypeI
		R: GTGGTTGTGGAGGAGAAGGA			
12	MtTFSSR-32	F: TCAGGATGTTTCCCATCCAT	4	0.69	ARF
		R: GCTGCTGTTGCTGTTGTTGT			
13	MtTFSSR-35	F: TTGTGGCTTTGCATATTGGA	4	0.70	C2H2_Zn
		R: GGATCTGTGCAGGAGTTGGT			
14	MtTFSSR-41	F: TCCCTACAGCAGGAGGTGAT	7	0.83	(TCA)5
		R: GATGCTCAGAACCAGCATGA			
15	MtTFSSR-42	F: CTGTGATGCCTTCTTCCACA	3	0.46	(R1)R2R3_MYB
		R: TTTCCCCAAGTATAGCTACCG			
16	MtTFSSR-43	F: ATGGCTGCTTTGTTACCTGG	3	0.22	(R1)R2R3_MYB
		R: TGTTGGGGATAAAGGGTGAA			
17	MtTFSSR-46	F: TCAAATTCACGTGCAGGAAG	4	0.69	C3H-TypeI
		R: TCATGAGCAGCCACAATCTC			
18	MtTFSSR-51	F: TCCTCAACCAACCACTTCCT	2	0.33	AP2_EREBP
		R: TCTCTGATACCCATTTGCCC			
19	MtTFSSR-52	F: GCCAAGCTGTTTCTTCTTCG	4	0.49	AP2_EREBP
		GTCTTCAAGCCGAAAACTCG			
20	MtTFSSR-55	F: GTCAAGGTGGTGGCTTTGAT	5	0.68	bHLH
		R: TCAATCTTGAATTGCCCCTC			
21	MtTFSSR-56	F: ATTGAGTTTTACCGGGGGAG	4	0.61	bHLH
		R: CGCATTGAGGCAATGTAGAA			
22	MtTFSSR-58	F: TGCAAATTACACCTTTGACCC	4	0.63	GARP_G2-like
		R: TCAAAAGGTGGTTGTGGTTG			
23	MtTFSSR-61	F: TGAGGAAGGTTCCAAGGATG	3	0.08	WRKY_Zn
		R: ATCATGTTAGCCTCGGATCG			
24	MtTFSSR-64	F: TAATGGGAGGAACATGCACA	4	0.49	C2C2_Zn-GATA
		R: AAGAGCGACGGTTTCGTTTA			
25	MtTFSSR-65	F: TCCACTTGAAGTCAACGCAG	2	0.33	AP2_EREBP
		R: GCTGACCAAACCCTTGACAT			
26	MtTFSSR-66	F: CAGCAGTACTGGCAATGATGA	3	0.52	NAC
		R: CTTCCAAAGTTCCATGTGGC			
27	MtTFSSR-70	F: TTCAAGACCGTCTCGGCTAC	7	0.84	TCP
		R: TGATGATTGTTCTGCTGCAA			
		Average	4.41	0.60	

## 3. Experimental Section

### 3.1. Plant Material and DNA Isolation

The leguminous species barrel medic (*Medicago truncatula* A17), chickpea (common vetch cultivar Lanjian 3), and alfalfa (*Medicago sativa* cultivar UC-1465) and the non-leguminous species tobacco (*Nicotiana tabacum* cv. Samsun NN), rice (*Oryza sativa* cv. Kitaake), and *Arabidopsis thaliana* ‘Columbia’ were used to examine the transferability of TFGM markers developed in this study. Genomic DNA was extracted from leaf material of greenhouse plants using a CTAB protocol as described previously [26]. A total of 44 alfalfa accessions (Table 3) were collected from the United States Department of Agriculture National Plant Germplasm System (NPGS) and the Institute of Animal Science, Chinese Academy of Agricultural Sciences (IAS-CAAS) in Beijing for genetic diversity analyses. Young leaves of 40 individual field plants from each accession were bulked as one sample and used for genomic DNA isolation as described above. The DNA quality and quantity were checked in 1% agarose gels and a NanoDrop ND1000 instrument (Thermo Scientific, Waltham, MA, USA), respectively. The DNA was normalized to 25 ng/µL for further use.

### 3.2. Identification of SSR and Primer Design

A total of 1467 TF coding sequences of *M. truncatula* were downloaded from LegumeTFDB [27] and used for identification and localization of SSRs by using a Perl 5 script (*MISA*, MIcroSAtellite identification tool). The minimum length criteria were defined as 10 and six repeat units for mononucleotide and dinucleotide repeats, respectively, and five repeat units for trinucleotide, tetranucleotide, pentanucleotide and hexanucleotide repeats. The maximum interruption between two SSRs was 100 base pairs (bp). Once SSRs had been identified from the TF sequences, flanking primers to SSRs were designed using Primer3 software in a batch modus manner with the help of Perl 5 interface modules [12]. The parameters for the primer design were as follows: amplicon size, 100–350 bp; primer length, 18–27 bases with 20 as the optimum; annealing temperature, 57–63 °C with the optimum of 60 °C; GC content, 45%–50%.

To determine the novelty of the TFGM markers developed in the present study, a stand-alone BLASTN (Basic Local Alignment Search Tool, http://blast.ncbi.nlm.nih.gov) search for the TF sequences used for TFGM markers development was performed against the 3828 *M. truncatula* EST sequences (as query, *E*-value = 10^−^^5^) previously reported in EST-SSR markers development [12]. Previously published and new TFGM SSR markers are both reported in this study for comparison.

### 3.3. Functional Annotation

Functional annotation of transcription factor genes based on Gene Ontology terms (GO) was analyzed by Blast2GO [28] and WEGO software [29].

### 3.4. PCR Amplification 

PCR amplifications were conducted in a final volume of 20 µL containing 50 ng template DNA, 1× PCR buffer, 2.0 mM MgCl_2_, 2.5 mM dNTPs, 4 µM each primer, and 0.8 unit of *Taq* polymerase (TaKaRa, Dalian). The PCR reaction cycling included 4 min at 94 °C, 35 cycles of 30 s at 94 °C, 35 s at 60 °C, and 1 min at 72 °C, with a final extension step of 5 min at 72 °C. Denatured PCR products were subjected to electrophoresis on 6.0% polyacrylamide gels, and the banding patterns were visualized using silver staining [2]. At least two independent PCR amplifications were performed for each primer.

### 3.5. Cross-Species Amplification

To assess the transferability of TFGM markers, we tested their amplification in leguminous and non-leguminous (as described in the plant material section) species, using PCR as described above.

### 3.6. Genetic Diversity Analysis

The SSR profiles (alleles) in a binary format were scored as present (1) or absent (0) and used for the genetic relationships determination among the different alfalfa accessions. Only specific bands that could be unambiguously scored across all alfalfa accessions were used in this study. Polymorphism information content (PIC) was calculated by PIC CALC 0.6 [15]. A dendrogram was constructed based on the genetic identify matrix using the unweighted pair group mean algorithm (UPGMA) of NTSYSpc software [30].

## Supplementary Materials

Supplementary materials can be accessed at: http://www.mdpi.com/1420-3049/20/05/8759/s1.
