# Supplementary material for: Development and Characterization of Transcription Factor Gene-Derived Microsatellite (TFGM) Markers in Medicago truncatula and Their Transferability in Leguminous and Non-Leguminous Species
Source: Molecules. 2015 May 15;20(5):8759–71. doi: 10.3390/molecules20058759 (PMC6272326; doi:10.3390/molecules20058759)
Supplement: Supplementary file 1 [file molecules-20-08759-s002.zip › Supplementary Files/Figure S1.pdf]

Supplementary Material

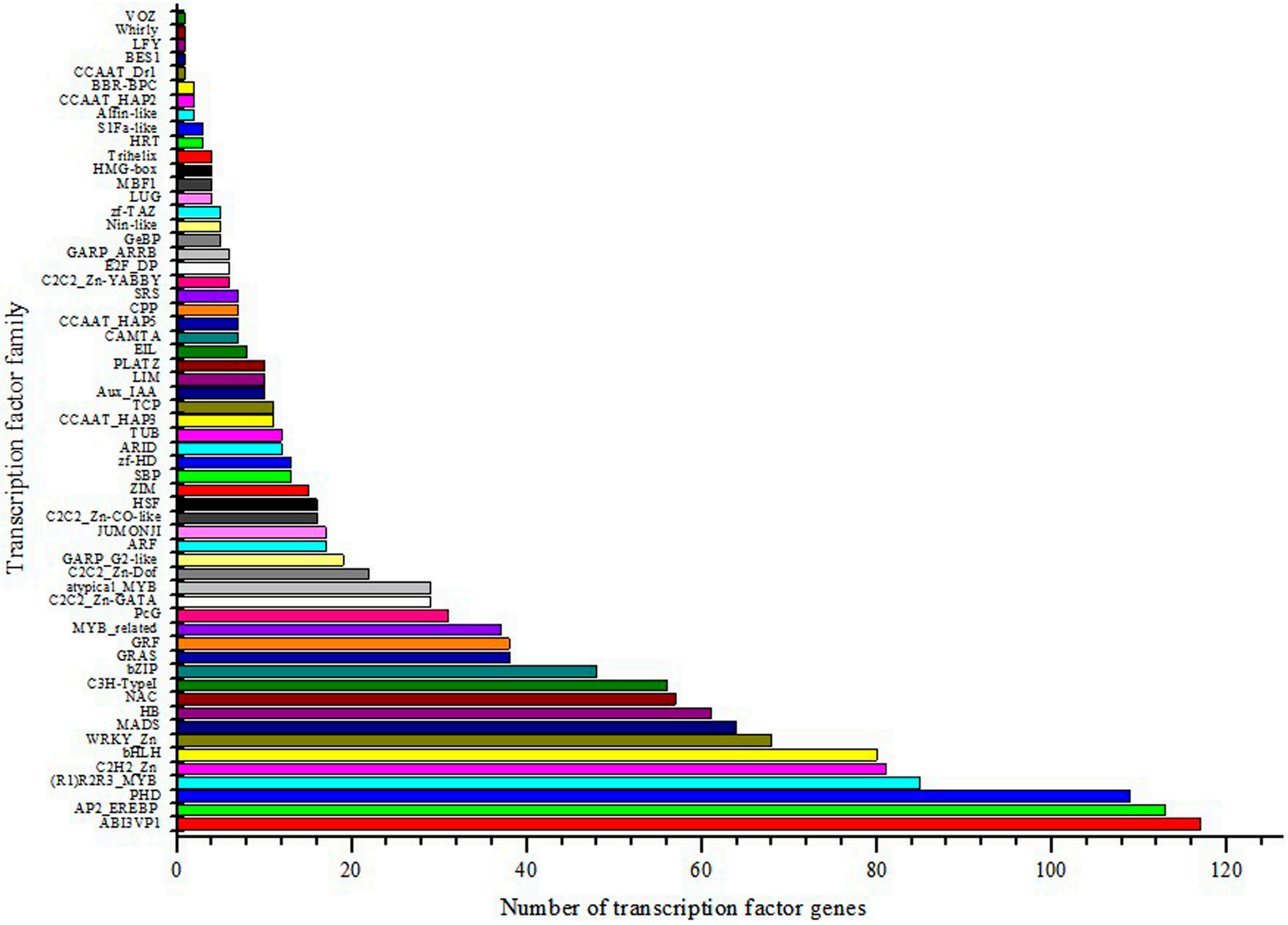

Figure S1. The number of genes in *M. truncatula* transcription factor families.
